# Supplementary material for: Evidence of structural discontinuities in the inner core of red-giant stars
Source: Nat Commun. 2022 Dec 16;13:7553. doi: 10.1038/s41467-022-34986-z (PMC9758139; doi:10.1038/s41467-022-34986-z)
Supplement: Supplementary file 1 — Supplementary Information [file 41467_2022_34986_MOESM1_ESM.pdf]

SUPPLEMENTARY INFORMATION

Evidence of structural discontinuities in the inner core of red-giant stars

Vrard et al.

October 20, 2022

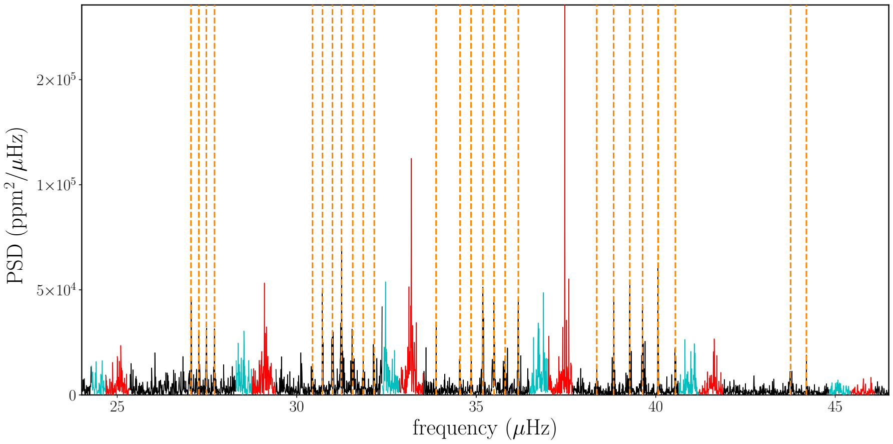

Supplementary Figure 1: Power density spectra (PSD) as a function of frequency for the oscillation spectrum of the star KIC3544063. The red and cyan colored-part of the oscillation spectra correspond, respectively, to the $\ell = 0$ and $\ell = 2$ modes. The dashed orange lines represent the frequency positions of the identified mixed-modes following the mode fitting. Source data are provided as a Source Data file.

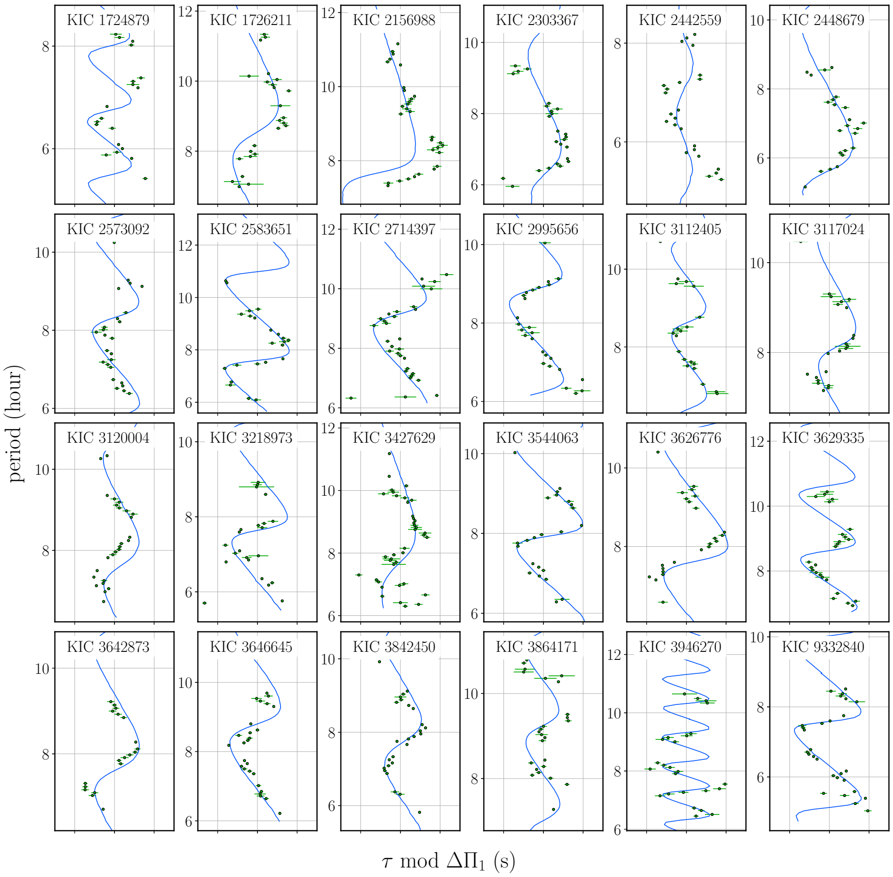

Supplementary Figure 2: Observed mixed mode periods (green dots) as a function of the stretched periods (τ) modulo $\Delta\Pi_1$ in an échelle diagram for the sample of stars (24) with identified glitches. The blue line represents the best fitted model to the observed mixed mode pattern. The star KIC2156988 was excluded from the final sample due to an unreliable fit (see Core glitches identification and characterization subsection). Source data are provided as a Source Data file.
