# Supplementary material for: Evidence of structural discontinuities in the inner core of red-giant stars
Source: Nat Commun. 2022 Dec 16;13:7553. doi: 10.1038/s41467-022-34986-z (PMC9758139; doi:10.1038/s41467-022-34986-z)
Supplement: Supplementary file 2 [file 41467_2022_34986_MOESM3_ESM.odt]

File Name: Supplementary_Data_1

Description: 1 additional table containing the physical parameters of the sample of stars in the study.

It contains 9 columns, respectively: Kepler Identification Number (KIC), large separation (∆ν  in μHz), 1-sigma error bars on the large separation ∆ν (in μHz), period spacing ∆Π1 (in s), 1-sigma error bars on the period spacing ∆Π1 (in s), seismic stellar mass in solar masses, 1-sigma error bars on the seismic mass in solar masses, stellar metallicity (in dex), 1-sigma error bars on the metallicity (in dex)

File Name: Supplementary_Data_2

Description: 24 files containing the mode frequencies of the 24 stars showing core glitches. The files are named following 'freqKIC' + Kepler Identifying Number of the star + '.txt'

Each file contains 4 columns, respectively: mixed-mode frequencies (ν in μHz), stretched periods modulo the gravity-mode period spacing (τ mod ∆Π1 in s), 1-sigma error bars on the mode frequencies (in μHz), 1-sigma error bars on the stretched periods modulo the gravity-mode period spacing (in s)
